# Risk of hyperkalemia in patients with moderate chronic kidney disease initiating angiotensin converting enzyme inhibitors or angiotensin receptor blockers: a randomized study

**DOI:** 10.1186/1756-0500-6-306

**Published:** 2013-08-01

**Authors:** Eugenia Espinel, Jorge Joven, Iván Gil, Pilar Suñé, Berta Renedo, Joan Fort, Daniel Serón

**Affiliations:** 1 Servei de Nefrologia, Hospital Vall d’Hebron, Universitat Autònoma, Barcelona, Spain; 2 Unitat de Recerca Biomèdica (URB-CRB), Institut d’Investigació Sanitària Pere Virgili, Universitat Rovira i Virgili, Reus, Spain; 3 Servei de Farmacia, Hospital Vall d’Hebron, Universitat Autònoma, Barcelona, Spain

## Abstract

**Background:**

Angiotensin-converting enzyme inhibitors and angiotensin II receptor blockers are renoprotective but both may increase serum potassium concentrations in patients with chronic kidney disease (CKD). The proportion of affected patients, the optimum follow-up period and whether there are differences between drugs in the development of this complication remain to be ascertained.

**Methods:**

In a randomized, double-blind, phase IV, controlled, crossover study we recruited 30 patients with stage 3 CKD under restrictive eligibility criteria and strict dietary control. With the exception of withdrawals, each patient was treated with olmesartan and enalapril separately for 3 months each, with a 1-week wash-out period between treatments. Patients were clinically assessed on 10 occasions via measurements of serum and urine samples. We used the Cochran–Mantel–Haenszel statistics for comparison of categorical data between groups. Comparisons were also made using independent two-sample *t*-tests and Welch’s *t*-test. Analysis of variance (ANOVA) was performed when necessary. We used either a Mann–Whitney or Kruskal-Wallis test if the distribution was not normal or the variance not homogeneous.

**Results:**

Enalapril and olmesartan increased serum potassium levels similarly (0.3 mmol/L and 0.24 mmol/L respectively). The percentage of patients presenting hyperkalemia higher than 5 mmol/L did not differ between treatments: 37% for olmesartan and 40% for enalapril. The mean e-GFR ranged 46.3 to 48.59 ml/mint/1.73 m^2^ in those treated with olmesartan and 46.8 to 48.3 ml/mint/1.73 m^2^ in those with enalapril and remained unchanged at the end of the study. The decreases in microalbuminuria were also similar (23% in olmesartan and 29% in enalapril patients) in the 4 weeks time point. The percentage of patients presenting hyperkalemia, even after a two month period, did not differ between treatments. There were no appreciable changes in sodium and potassium urinary excretion.

**Conclusions:**

Disturbances in potassium balance upon treatment with either olmesartan or enalapril are frequent and without differences between groups. The follow-up of these patients should include control of potassium levels, at least after the first week and the first and second month after initiating treatment.

**Trial registration:**

The trial EudraCT “2008-002191-98”.

## Background

The rate of raised serum potassium concentration in hospitalized patients and in admissions to emergency departments is high and may represent an ominous marker of increased risk of death [1]. This is more common among patients with impaired renal function and defects in the excretion of renal potassium, with some associated clinical conditions and treatment with a growing list of drugs [2-7]. Although there is considerable inter-individual variation in susceptibility, hyperkalemia may be responsible for alterations in the excitatory capacity of the heart conduction system and is consequently associated with severe arrhythmogenesis and fatal consequences [8,9].

The incidence of hyperkalemia is quite low in patients with normal renal function: >2% but increases from 2% to 42% as the GFR diminishes to 20 ml/min 1.73/m^2^[10]. There are multiple triggering factors in chronic kidney disease (CKD) patients, but a significant proportion of episodes of hyperkalemia are attributed to the use of drugs taken to alleviate concomitant hypertension, especially angiotensin-converting enzyme inhibitors (ACEIs) and angiotensin receptor blockers (ARBs) as they inhibit the renin-angiotensin system and cause a reduction in serum aldosterone [11]. It has been also described that hyperkalemia develops in approximately 10 percent of outpatients within a year of ACEIs being prescribed [12]. Furthermore, in six separate clinical trials of more than 1500 people with CKD, increased levels of 0.3-0.6 mmol/L were detected in the ACEI randomized patients [7]. This increase in serum potassium led to discontinuation of ACEI therapy in 1.2 to 1.6% of patients in any given trial.

Both ACEIs and ARBs are widely included in clinical guidelines to manage hypertension and other risk factors associated with the course of atherosclerosis [13-15] and may significantly delay the progression of renal damage in patients with chronic kidney disease [16-21]. Therefore, nephrologists face a paradoxical and clinically significant challenge in this realm because those patients who would benefit most from treatment with ACEIs or ARBs are precisely those with the highest risk of adverse effects. In addition, in these patients any prediction of potentially dangerous potassium disturbances is complicated by the consequences of a non-controlled diet, concomitant drugs and other associated chronic diseases. Consequently, safety issues regarding the use of these drugs in patients with renal insufficiency and in those with moderate CKD are not yet completely established [22,23]. The real incidence of hyperkalemia as a result of these treatment regimes is not well known because available evidence is difficult to interpret due to the influence of confounding factors [24-26], which is particularly evident in patients with CKD [27]. Despite the lack of clear evidence some authors have stated that the increase in serum potassium is less pronounced during therapy with ARBs and that the risk of hyperkalemia is higher in patients treated with ACEIs [7,23].

Data supporting potential differences between ACE inhibitors and ARBs in serum potassium concentrations come from clinical trials that compare the effect of an ACEI to an ARB on renal function in people with heart failure. These trials demonstrated a lower incidence of hyperkalemia in patients randomly assigned to an ARB treatment compared with those randomized to ACEI treatment. In these studies there was a similar proportion of patients with CKD but they were receiving, at the same time, not only other different potassium-influencing drugs (diuretics, betablockers or potassium supplements) [24,28], but at different and non-specified doses.

We believe to demonstrate that one drug or another produces more or less hyperkalemia it is indispensable that this be the only potassium-influencing medication utilized. So currently there is little clear evidence for assuming differences between equipotent doses of ARB and ACEI to cause hyperkalemia.

Hence, our study is designed in this way, so that the only potassium-influencing drugs which our patients received were those permitted in the trial. All our patients had the same level of CKD and they followed a standardized potassium diet.

Bearing in mind the large amount of patients indicated to receive ACEIs or ARBs, the risk of associated CKD (heart failure, advanced age, etc.), as well as the risk of hyperkalemia, we believed that it would be of great interest to learn whether one of these medications produce less hyperkalemia than the other.

In this study we challenge this assumption by assessing the effect of standard-of-care treatment with olmesartan and enalapril, as representatives of ARBs and ACEIs respectively, on the incidence of hyperkalemia in patients with stage 3 CKD.

## Methods

It was designed as a randomized, double-blind, phase IV, controlled, crossover study.

**Figure 1:**
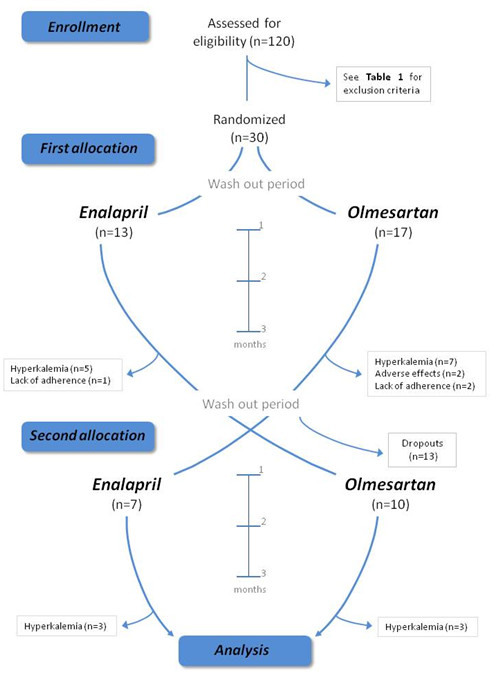
Flow diagram of participants through each stage of the trial indicating for each group the number of participants assigned that received intended treatment and were analyzed for the primary outcome.

Among patients attending our outpatient clinic for management of CKD in a tertiary care teaching hospital, patients with stage 3 CKD, i.e. estimated GFR (eGFR) between 30 and 60 ml/min/1.73 m^2^, stable clinical condition, aged 18–75 years, serum potassium concentration <5 mmol/L and blood pressure (BP) ranging between 130/80 mmHg and 180/100 mmHg, were considered for inclusion (n = 120). The use of calcium channel blockers or alpha-adrenergic blockers was not an exclusion criterion. For enrollment, strict exclusion criteria were established to exclude the presence of confounding factors that could alter the incidence of potassium disturbances during the treatment period as outlined in Table 1. No patient with a known arterial renal stenosis was included. The attending physicians identified 34 eligible patients, 4 of whom were discarded during the screening visits due to failure to follow a balanced diet that was relatively low in sodium and contained 80–90 mmol/day of potassium as assessed via repeated measurement of electrolytes in 24-h urine samples. To fulfill restrictive eligibility criteria and strict dietary control patients were also instructed not to ingest salt substitutes or herbs. Finally we recruited 30 patients with stage 3 CKD who were informed about the trial and invited to participate and sign the informed consent approved by our institutional review board. Patients received olmesartan and enalapril sequentially, (Figure 1) and we chose the two-period design for simplicity. Raised serum potassium levels and lack of adherence were defined as reasons for withdrawal.

**Table 1:** Criteria for exclusion reported during the enrollment period

Declined to participate	(n = 26)	Secondary arterial hypertension	(n = 5)
Previous allergic reaction to ACEIs	(n = 3)	Previous allergic reaction to ARBs	(n = 2)
Active disease in other organs	(n = 6)	Previous gastrointestinal surgery	(n = 1)
Diseases causing malabsorption	(n = 1)	Recent (1 year) ischemic episodes	(n = 9)
History of ventricular arrhythmias	(n = 2)	History of cardiac insufficiency	(n = 8)
Current prescription with diuretics	(n = 9)	Current prescription with potassium	(n = 4)
Current prescription with B-blockers	(n = 7)	Current prescription with NSAIDs	(n = 3)

***ACEIs***: Angiotensin-converting enzyme inhibitors, ***ARBs***: Angiotensin receptor blockers, ***NSAIDs***: nonsteroidal anti-inflammatory drugs, ***B-blockers:*** Beta-blockers.

This number was considered sufficient according to available data [23] and assuming a difference between groups exceeding 10%, an overall standard deviation of 20% and 80% power with a two-sided 5% significance level. We anticipated that dropouts and withdrawals would not influence the primary outcome, i.e. the proportion of patients in whom hyperkalemia was detected. We then proceeded according to Figure 1. Enrolled patients (20 men and 10 women) were born in our geographic area and all of them were Caucasian. There were no differences in age, BMI or other relevant variables (Table 2). Thus patients received olmesartan and enalapril sequentially in a controlled crossover, longitudinal design (Figure 1). We considered hyperkalemia as potassium level of 5.0 mmol/L or higher [10,29].

**Table 2:** Participants’ characteristics and values for selected variables before the first allocation

	**Olmesartan**	**Enalapril**	**P-value**
	**(n = 17)**	**(n = 13)**	
Age, years	60.2 (12.9)	59.9 (11.6)	NS
Female, *n* (%)	8 (47.1)	2 (15.4)	<0.0001
BMI, kg/m^2^	27.7 (6.2)	27.41 (4.1)	NS
Systolic blood pressure, mm Hg	134.8 (4.2)	140.1 (4.2)	NS
Diastolic blood pressure, mm Hg	78.7 (1.7)	76.2 (1.8)	NS
Dyslipidaemia, *n* (%)	6 (35.3)	5 (38.5)	NS
Serum creatinine, mg/dl	1.65 (0.07)	1.60 (0.06)	NS
Glomerular filtration rate, ml/min/1.73 m^2^	42.24 (2.0)	46.2 (1.9)	NS
Plasma renin activity, ng/ml/h	1.13 (1.12)	1.30 (1.46)	NS
Plasma aldosterone, ng/dl	30.6 (4.0)	27.1 (5.5)	NS
Diabetic patients, *n* (%)	1 (5,8)	2(15)	NS

*BMI:* Body mass index, *NS:* no significant.

Values are expressed as mean ± SE of the mean.

We withdrew ACEIs, ARABs, betablockers, diuretics and any drug which could influence potassium levels 15 days before performing the baseline analyses. After that we randomized the patients to receive either: 10 mg of olmesartan or 10 mg of enalapril for one week, after which we performed an analytic determination. At that point patients with potassium levels <5 mmol/L were instructed to increase the dose to 20 mg/day in week 3, with an analytic control at week 4. Following, that controls were performed at weeks 8 and 12. Once this phase was finished and after a 7–10 day wash-out period, the patients were prescribed the alternative drug and repeated this itinerary for three months more. Any patient with potassium >5 mmol/L was withdrawn from the trial. Those patients withdrawn from the first phase underwent a 7–10 day wash-out period and, after ensuring their potassium levels were normal, were then transferred to the second phase.

Each patient was visited 10 times throughout the study for reinforcement and to avoid severe complications. Health care providers and participants were blinded to the drug assignment. The members of staff responsible for the intervention were instructed to temporarily stop the medication if hyperkalemia, changes in renal function or lack of adherence to the diet were detected. This was achieved with the independent contribution of pharmacists who were also responsible for dispensing the drugs in numbered bottles to conceal the allocation sequence, simple randomization using a computerized random number generator and storage of the allocation list. These procedures resulted in unbalanced allocation (n = 17 for olmesartan and n = 13 for enalapril). After the wash-out period at the crossover stage, some patients refused to continue the study, alleging the requirement for excessive commitment. Secondary measurement outcomes included potentially affected variables. Blood pressure and eGFR were measured as described elsewhere [13,30]. Adherence to medication and diet was assessed using the Morisky-Green test and the brief medication questionnaire [31,32]. Whilst no data is available comparing equipotent doses of olmesartan (half life 12 hours) with enalapril (half life 11 hours), there are other studies comparing 5 mg of ramipril with 20 mg of enalapril or 5 mg of ramipril with 20 mg de olmesartan, meaning that these doses of enalapril and olmesartan could be considered comparable [33,34].

Routine analyses at each visit included creatinine, potassium, sodium and osmolarity in serum and albumin (microalbuminuria), creatinine, sodium and potassium in urine. At the beginning and the end of two periods of treatment (visits 1, 5, 6 and 10), plasma renin activity and aldosterone were also measured. The trial was according to the Helsinki declaration and was authorized by the local ethics committee and by the Spanish Agency for Medicines and Medical Products (AGEMED) which provided support and authorized the trial EudraCT “2008-002191-98”.

All analyses were performed according to standard procedures using automatic analyzers. Allocation concealment was extended to the laboratory personnel. Unless otherwise stated, variables are expressed as mean and standard error of the mean. We used the Cochran–Mantel–Haenszel statistics for comparison of categorical data between groups, particularly the proportion of patients with hyperkalemia. Comparisons were also made using independent two-sample *t*-tests, in some cases with unequal sample sizes and unequal variance (Welch’s *t*-test). To avoid multiple two-sample t-tests and the increased chance of committing a type I error, analysis of variance (ANOVA) was performed when necessary. We used either a Mann–Whitney or Kruskal-Wallis test if the distribution was not normal or the variance was not homogeneous. A two-tailed P < 0.05 was considered statistically significant. Individual missing data were not imputed in an expectation-maximization algorithm. Statistical analyses were performed using the Statistical Package for the Social Sciences, version 18.0 (SPSS Inc., Chicago, Il, USA).

## Results

During the first period, 38% of patients receiving enalapril developed hyperkalemia, all during the first week. Hyperkalemia in the olmesartan group was detected in 41% of patients at different time-points (weeks 1 (n = 3), 4 (n = 3) and 8 (n = 1)). At the end of the first period, 13 patients declined to continue for various reasons, confirming the high rate of attrition in studies with designs that require strong commitment, continuous control of diet and other confounding factors. We considered that 7–10 days of wash out was adequate because potassium and aldosterone levels in all patients had returned to their basal before beginning the second phase. During the second period, 42% of patients in the enalapril group developed hyperkalemia, and thus were withdrawn from the study at weeks 4 (n = 1) and 8 (n = 2). A similar percentage of those treated with olmesartan also developed kyperkalemia at the same time-points. Therefore, 27 patients fully participated in the olmesartan arm and 20 in the enalapril arm, resulting in unbalanced groups that prevented inferences with respect to secondary outcomes and further analysis of association between the presence of hyperkalemia and other measured variables. Mild adverse effects were reported in both groups, n = 16 for olmesartan and n = 10 for enalapril. We had two diabetic patients in the enalapril group and one in the olmesartan group (Table 2). One out of three of the diabetic patients included in the study showed raised potassium levels with both drugs. Two patients showed hyperkalemia with olmesartan and not with enalapril and likewise 2 patients showed hyperkalemia with enalapril and not with olmesartan.

**Figure 2:**
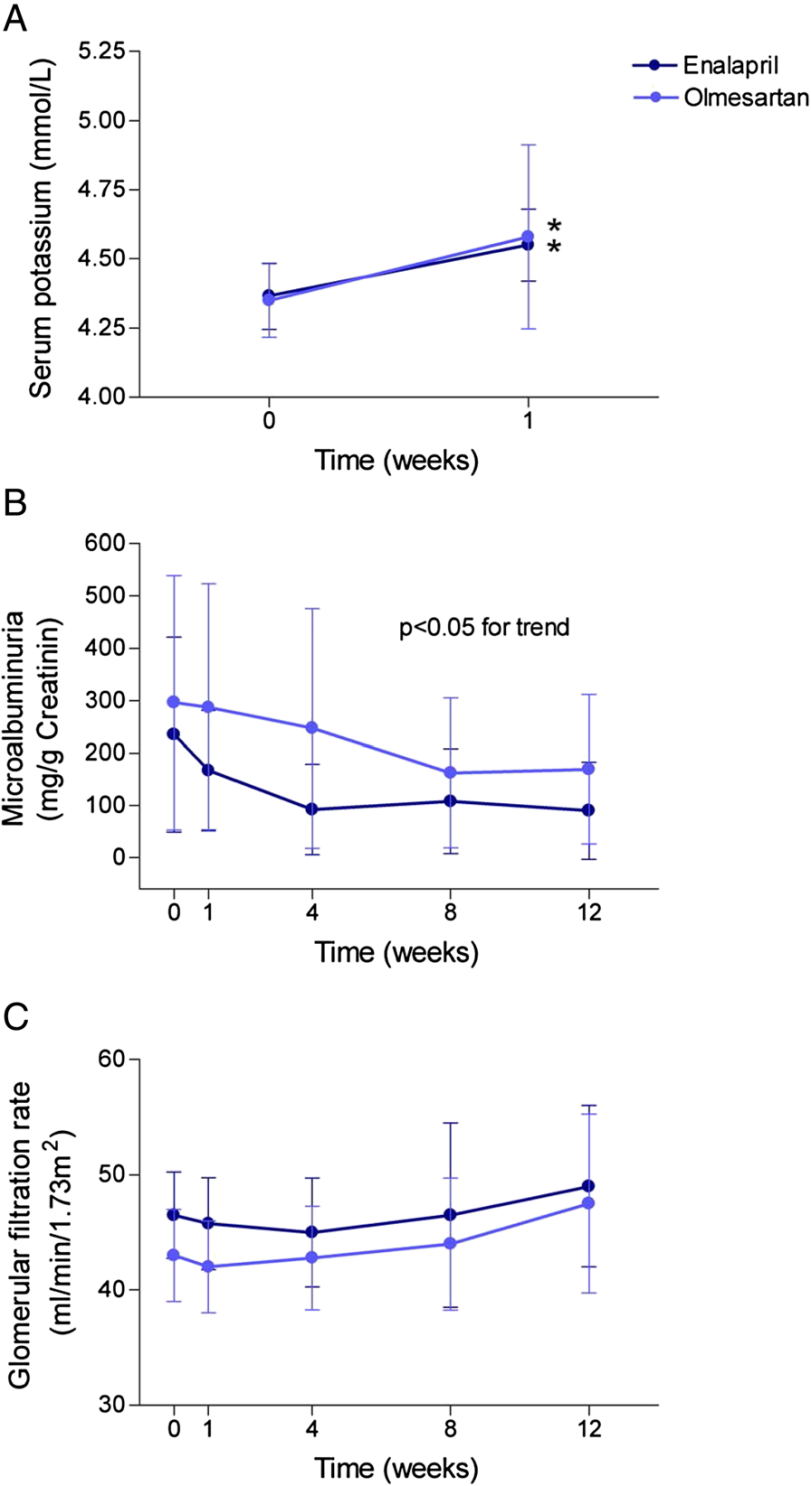
**The increase in serum potassium concentration during the first week of treatment was significant and similar for enalapril (n = 20) and olmesartan (n = 27). (A)** The effects of both drugs in microalbuminuria **(B)** and glomerular filtration rate **(C)** during the trial were also similar between groups. Valid results for e-GFR were obtained for enalapril: n = 22,16,14,14 and for olmesartan: n = 24,24,16,11 for measurements at t = 0, 1, 4, 8 and 12 weeks, respectively. Valid results for microalbuminuria were obtained for enalapril: n = 17,14,10,10 and for olmesartan: n = 24,22,14,12 for measurements at t = 0, 1, 4, 8 and 12 weeks, respectively. *p < 0.05.

Of note, there was no significant increase in serum potassium with either olmesartan or enalapril in 50% of patients. There were no significant differences in the percentage of patients with hyperkalemia with respect to the treatment group (37% for olmesartan and 40% for enalapril). However, there was a significant (p < 0.05) and immediate (1 week) increase in serum potassium levels with respect to baseline values in both treatments; 10.5% for olmesartan (from 4.35 ± 0.05 to 4.59 ± 0.05 mmol/L) and 10.7% for enalapril (from 4.30 ± 0.06 to 4.60 ± 0.09 mmol/L) (Figure 2). This trend only remained significant during the first 8 weeks. Olmesartan patients showed potassium levels of up to 5.1 mmol/L: 2 patients in week 1, up to 6.5 mmol/L: 2 patients in week 4 and up to 5.1 mmol/L: 1 patient in week 8. Patients treated with enalapril showed potassium levels of up to 5.5 mmol/L: 1 patient in week 1, up to 5.4 mmol/L: 1 patient in week 4 and up to 5.2 mmol/L: 2 patients in week 8.

That is to say, up to week 8, 3 out of 30 (10%) patients who were treated with these drugs did not show potassium levels over 5 mmol/L. Only two patients, one in each treatment group, reached potassium levels above 5.5 mmol/L, and the highest figure was 6,4 mmol/L in a diabetic patient.

In enalapril treated patients baseline systolic BP was of 135.78 ±16 mmHg and of 124 ±13 mmHg at the end p < 0.05, baseline diastolic BP was 79.7 ± 6.8 mmHg and 74 ±10 mmHg at the end p = 0.09. In olmesartan treated patients baseline systolic BP was 136.33 ± 17.9 mmHg and 131.13 ± 13 mmHg at the end p = 0.4, diastolic baseline BP was 79 ± 7 mmHg and 78 ± 9 at the end p = 0.7. It is true that the drop in systolic BP was significant in enalapril patients with regards to baseline, however the differences were not significative when we compared the systolic and diastolic BP at baseline between olmesartan and enalapril patients and systolic and diastolic BP at the end of the study.

In that sense we should say that our objective was not BP control but to maintain the ACEI/ARB doses in order to perform strict evaluation of potassium levels. The patients were receiving different doses of doxazosine or calcium-channel-blockers at baseline and in many cases their BP was already treated and controlled before beginning the trial. If the patient required a drug to lower BP we did this with different doses of doxazosine or calcium-channel-blockers. Therefore, we believe that it is not possible to draw conclusions on BP evolution.

Furthermore, among those terminating the study there were no significant differences in urinary potassium with respect to baseline values (79.6 ± 9.4 vs. 81.1 ± 8.1 mmol/L for olmesartan and 81.7 ± 15.2 vs. 62.7 ± 6.5 mmol/L for enalapril) or appreciable changes in daily urine sodium elimination. Similar trends were observed for the transtubular potassium gradient (6.7 ± 0.4 vs. 6.75 ± 0.5 for olmesartan and 6.8 ± 0.6 vs. 6.21 ± 0.7 for enalapril). The baseline serum osmolarity was within normal levels. At 12 weeks we observed a trend towards higher plasma renin activity in both arms that did not reach statistical significance (1.13 ± 1.12 vs. 1.56 ± 1.77 and 1.30 ± 1.46 vs. 1.61 ± 1.83 ng/ml/h for olmesartan and enalapril respectively) and lower plasma aldosterone that was only significant (p < 0.05) for enalapril (27.6 ± 3.8 vs. 18.7 ± 2.9 ng/dl).

Microalbuminuria, which was tested as a urine spot: mg albumin/gr creatinine ratio, was 278 ± 134 and 151 ± 119 at baseline for olmesartan and enalapril patients respectively, which had diminished to 213 ± 90 and 106 ± 59 respectively (Figure 2) at the end of the 4^th^ week. That represents an overall decrease in microalbuminuria concentrations of 23% and 29% for the olmesartan and enalapril groups respectively. This decrease only reached statistical significance when compared with ANOVA indicating a detectable trend towards a positive effect. We found no further decrease of microalbuminuria in the following controls (Figure 2).

Finally, baseline eGFR of olmesartan patients went from 46.3 ± 3.7 to 48.59 ± 4.3 ml/mint/1.73 m^2^ at the end, and the baseline eGFR of enalapril patients went from 46.8 ± 3.1 to 48.3 ± 4.3 ml/mint/1.73 m^2^ at the end. The e-GFR underwent a slight decrease in the first weeks, recovering at the end of the study (Figure 2).

## Discussion

Sudden cardiac arrest and arrhythmia are prominent causes of death among patients with chronic kidney disease and in most cases the patient’s potassium level is associated with the risk of death [35]. Therefore, it is clinically advisable to carefully monitor these patients, to ensure proper dietary management and to avoid potentially deleterious drugs. According to our data, in this CKD stage 3 group, olmesartan and enalapril increased potassium levels at a mean of 0.3 mmol/L, which was greater than 5 mmol/L in 40% of patients. The clinical implications are relevant because potassium levels require continuous surveillance and because these drugs are effective in retarding the progression of renal disease, especially when this is moderate and requires strict control of blood pressure [36]. A possible clinical difference between ACEIs and ARBs in terms of their effect on serum potassium was initially described [7,24,28], but modifications to drugs in these categories are continuously being incorporated into the therapeutic armamentarium, with possible, unexplored effects. A common problem is that trials do not appreciate the effect of stringent criteria for withdrawal and the strict control of confounding factors [8,12,16]. There has been no previous clinical study comparing olmesartan and enalapril in stage 3 CKD patients. We continuously controlled sodium and potassium intake and excretion in order to limit possible changes in serum potassium levels to those due to the presence of the drugs assayed. We found no significant differences between olmesartan and enalapril in their capacity to cause hyperkalemia in patients with e-GFR >30 and <60 ml/min/1.73 m^2^ but confirmed that both drugs influence the potassium balance [7,26], decrease urinary albumin excretion by 25% at 1 month and maintain renal function. Additionally, we found that we should not expect changes in serum potassium levels due to these drugs in approximately half of patients with CKD in whom diet is controlled. As previously suggested this may be partially attributed to the unequal distribution of polymorphisms in the angiotensin-converting enzyme gene [37].

Differences between ACEIs and ARBs, however, were initially plausible considering their differential effect on the bradikinine metabolism and plasmatic renal flow [22,23]. Our findings of lower plasma aldosterone and greater anti-hypertensive effects with enalapril provide further support for such differences. There were no differential effects on serum potassium level in uncontrolled patients with normal renal function, as demonstrated in a comparison between lisinopril and candesartan [38]. Only one study exists, also well designed, which compared 80 mg of Valsartan with 10 mg of lisinopril in 18 stage 3 CKD patients and found that valsartan produces less hyperkalemia. The explanation could lie in the different half lives of these drugs (12 hours for lisinopril and 6 hours for valsartan) [39], which could mean valsartan had less effect on potassium levels. In this study there is no reference to the potassium intake [7].

There are no data to support a difference in potency between olmesartan and enalapril; they have similar half lives (11 hours for enalapril and 12 hours for olmesartan) [39] and animal models indicate that olmesartan and enalapril are equivalent [40]. Moreover, studies comparing ACEIs and ARBs have yielded similar results [36,41]. No differences have been reported between ACEIs [42], but olmesartan has been reported as the most potent ARB to date [43,44].

We were particularly careful to avoid differences in dietary potassium intake. We are aware that assessment may be biased by the type of ingestion and the methods of recall [45] and consequently we included continuous laboratory measurement of electrolyte balance. Lack of adherence was limited but caused some withdrawals. The intake of potassium in a free-diet varies between 42 and 270 mmol/day. Our restrictions resulted in similar individual daily urinary potassium and sodium excretion and no relevant differences between drugs, supporting our conclusion that 20 mg of olmesartan does not produce more hyperkalemia than 20 mg of enalapril in stage 3 CKD patients. It is difficult to know if these results can be attributed to the entire pharmacological class or specifically to these two drugs.

In fact both drugs increased serum potassium levels. This increase was not particularly high due to our selection criteria and exhaustive control but values higher than 5 mmol/L were found in 30–40% of patients studied, indicating a frequent concern at this stage that is probably more important in patients with more severe CKD and in patients with dual blockade of the renin-angiotensin system [4]. Clinicians should consider that in daily clinical practice, in the “real world”, patients are usually influenced by a free potassium diet and established or temporary conditions that may aggravate hyperkalemia. Hence the effect of prescribed drugs should be monitored frequently. If treatment is not readily withdrawn, as described previously [12], the incidence of severe hyperkalemia may increase, notably within the first year of treatment. This is particularly relevant as severe hyperkalemia is found in 1% of ambulatory patients and most with suboptimal follow-up and management; very moderate increases in serum potassium may also generate arrhythmia [10,46]. We also wish to underline that having “slightly” and sustained, higher levels of potassium, not affecting the electrical cardiac conduction, could cause other unknown effects in the long term. On the other hand, we believe that these drugs should be utilized when indicated, if well managed (diuretics, diet, etc.) to maintain adequate potassium levels**.** Bearing in mind that some patients showed hyperkalemia with one drug and not with the other, we believe that in those patients with indispensable indication, for example, heavy proteinuria, we could try the alternative drug.

It is well documented that CKD patients in whom treatment with ACEIs or ABRs is indicated should be frequently checked for serum potassium levels. The most usual recommendations indicate that this should be done 7–10 days after prescription [10].

Nevertheless, in the light of our results we believe that stage 3 CKD patients treated with these drugs should also be controlled at the end of month 1 and 2; After that point, patients should be controlled at the periods recommended at guidelines, to follow-up stable stage 3 CKD patients, and also when a concomitant disease appears. Whether this is a cost-effective recommendation remains to be ascertained.

## Conclusions

In conclusion, patients with stage 3 CKD are prone to disturbances in potassium balance upon treatment with either olmesartan or enalapril. Hyperkalemia was present in a significant number of patients and there were no relevant differences between drugs. The follow-up of these patients should include frequent measurement of potassium levels at least during the first 2 months of treatment.

## Abbreviations

ACEI: Angiotensin-converting enzyme inhibitors; ARB: Angiotensin receptor blocker; GFR: Glomerular filtration rate; CKD: Chronic kidney disease; e-GFR: Estimated-GFR; BP: Blood pressure.

## Competing interests

The authors declare that they have no competing interests.

## Authors’ contributions

EE conceived the study, and participated in its design, interpretation of data and coordination and helped to draft the manuscript. JJ was involved in interpretation of data and also helped to draft the manuscript. IG participated in coordination and interpretation of data. PS participated in the design of the study and performed the statistical analysis. BR took part in the sequence of randomization and participated in the design. JF participated in coordination and revising the manuscript for intellectual content. DS collaborated with revising the manuscript for intellectual content. All authors read and approved the final manuscript.
